# Clinical, hormonal and genetic characteristics of androgen insensitivity syndrome in 39 Chinese patients

**DOI:** 10.1186/s12958-020-00593-0

**Published:** 2020-04-28

**Authors:** Qingxu Liu, Xiaoqin Yin, Pin Li

**Affiliations:** Department of Endocrinology, Shanghai Children’s Hospital, Children’s Hospital Affiliated to Shanghai Jiao Tong University, Shanghai, 200062 People’s Republic of China

**Keywords:** Androgen insensitivity syndrome (AIS), Androgen receptor mutation, 46, XY DSD, Mutation

## Abstract

**Background:**

Abnormal androgen receptor (AR) genes can cause androgen insensitivity syndrome (AIS), and AIS can be classified into complete androgen insensitivity syndrome (CAIS), partial androgen insensitivity syndrome (PAIS) and mild AIS. We investigated the characteristics of clinical manifestations, serum sex hormone levels and AR gene mutations of 39 AIS patients, which provided deeper insight into this disease.

**Methods:**

We prospectively evaluated 39 patients with 46, XY disorders of sex development (46, XY DSD) who were diagnosed with AIS at the Department of Endocrinology of Shanghai Children’s Hospital from 2014 to 2019. We analysed clinical data from the patients including hormone levels and AR gene sequences. Furthermore, we screened the AR gene sequences of the 39 AIS patients to identify probable mutations.

**Results:**

The 39 AIS patients came from 37 different families; 19 of the patients presented CAIS, and 20 of them presented PAIS. The CAIS patients exhibited a higher cryptorchidism rate than the PAIS (100 and 55%, *P* = 0.001). There were no significant difference between the CAIS and PAIS groups regarding the levels of inhibin B (INHB), sex hormone-binding globulin (SHBG), basal luteinizing hormone (LH), testosterone (T), or basal dihydrotestosterone (DHT), the T:DHT ratio, DHT levels after human chorionic gonadotropin (HCG) stimulation or T levels after HCG stimulation. However, the hormone levels of AMH (*P* = 0.010), peak LH (*P* = 0.033), basal FSH (*P* = 0.009) and peak FSH (*P* = 0.033) showed significant differences between the CAIS group and the PAIS group. Twenty-one reported pathogenic and 9 novel AR mutations were identified. Spontaneous AR mutations were found in 5 AIS patients, and 21 patients inherited mutations from their mothers, who carried heterozygous mutations.

**Conclusions:**

Forty-six XY DSD patients with cryptorchidism and female phenotypes were highly suspected of having AIS. We demonstrated that CAIS patients could not be distinguished by their hormone levels alone. Compared with PAIS patients, CAIS patients exhibited higher basal FSH, peak FSH, and peak LH hormone levels but lower AMH expression. We identified 21 reported pathogenic AR mutations and 9 novel AR mutations that led to different types of AIS. Missense mutations were the major cause of AIS and mostly occurred in exon 7 of the AR gene. These findings provided deeper insight into the diagnosis and classification of AIS and will even contributed to its clinical assessment.

## Background

Androgen insensitivity syndrome (AIS) is an X-linked genetic disease that is commonly caused by 46, XY disorders of sex development (46, XY DSD) [1]. The human androgen receptor (AR) gene is located in the Xq11–12 region and exhibits 8 exons that encode a peptide of 920 aa in length [2]. There are three ligand-dependent transcription factors corresponding to the major functional regions of the AR gene. These regions can be activated by androgens and interact with other proteins [3]. The critical period of reproductive organ development is from 8 to 14 weeks of gestation in humans depending on the presence of androgens and a normal AR. Therefore, abnormal androgen secretion and a defective AR can affect the conversion of androgens. Common receptor defects in AIS patients result from mutations in AR genes. Most reports of AR mutations have focused on small families and have lacked hormonal analysis [4], while some reports addressing the clinical and hormonal characteristics of large groups do not reveal the underlying mutations. We retrospectively studied the clinical manifestations, genotypes and serum hormones of 39 AIS patients in whom AR gene mutations were identified to lay a foundation for better understanding AIS.

## Subjects and methods

### Subjects

Informed parental consent, patient consent and approval from the Hospital Ethics Committee were obtained before initiating the study. Thirty-nine patients with AIS, including 4 subjects from 2 families, were recruited for this study from the Department of Endocrinology, Shanghai Children’s Hospital, from 2014 to 2019. Thirty-eight of the patients were diagnosed with AIS under the age of 11, and 1 patient was diagnosed at the age of 13 years and 8 months. The patients met the following criteria for AIS: they exhibited a 46, XY karyotype, SRY (+), normal adrenal function, AR mutations and serum hormone levels consistent with AIS. We investigated the clinical manifestations, family history, related sex hormones and AR gene sequences of the patients. In our study, AIS was classified as either complete androgen insensitivity syndrome (CAIS) or partial androgen insensitivity syndrome (PAIS) according to the clinical manifestations.

### External genital phenotype evaluation

We evaluated external genital phenotypes according to the PRADER classification [5]. Phallus length was compared with the data from normal Chinese children. The phallus was placed in an extended state, and the length from the pubic symphysis to the top of the glans along the dorsal side of the phallus excluding the length of the foreskin was recorded as the length of the phallus. Cryptorchidism was confirmed by physical examination and ultrasound examination in line with the diagnostic criteria for paediatric cryptorchidism. Cryptorchidism was classified as intra-abdominal cryptorchidism or inguinal cryptorchidism according to the location of the testis.

### Hormonal analysis

To evaluate testicular function, all patients underwent gonadotropin-releasing hormone (GnRH) stimulation to assess hypothalamic-pituitary-gonadal (HPG) axis function and human chorionic gonadotropin (HCG) stimulation. Sex hormones including anti-Mullerian hormone (AMH), inhibin B (INHB), oestradiol (E2), sex hormone binding globulin (SHBG), basal testosterone (T), basal dihydrotestosterone (DHT), basal luteinizing hormone (LH), peak LH, basal follicle-stimulating hormone (FSH), peak FSH, DHT after human chorionic gonadotropin (HCG) stimulation and T after HCG stimulation were detected. Serum LH and FSH concentrations were tested with LH and FSH detection kits (Beckman Coulter) and measured with an automatic immunoluminescence analyser (UnicelDxI 800). Serum AMH and INHB were detected in solid-phase sandwich enzyme-linked immunosorbent assays (ELISAs) purchased from Guangzhou Kangrun Biotechnology Co., Ltd. E2, T and DHT were tested by ELISA and measured with a USA Polar ELx800 microplate reader.

### AR gene analysis

Genomic DNA was obtained from peripheral blood leukocytes via a salting out procedure [6]. Exons 1–8 of the AR gene were amplified, and Sanger sequencing was used to screen for mutations. We searched the mutation assessment database within the human gene mutation database (http://www.ncbi.nlm.nih.gov) and the androgen mutation database (http://androgendb.mcgill.ca). PolyPhen-2 software was applied to perform pathogenicity analysis of amino acid changes caused by mutations.

### Statistical analysis

SPSS 25.0 software (manufactured by International Business Machines Corporation) was used to analyse these data. The nonparametric data were analysed with the Mann–Whitney U test and are presented as median values. The normally distributed data were analysed with the t-test and are presented as the mean ± S.D. Spearman correlation analysis was used for correlation analysis. Fisher’s test was applied to compare the incidence of cryptorchidism. A *P*-value < 0.05 was considered to indicate a significant difference, which was indicated as follows;*P < 0.05, **P < 0.01.

## Results

### Clinical manifestations

The data analysis showed that 20 (51.28%) patients exhibited a complete female external genital phenotype (PRADER grade 0) among the 39 patients with AIS, and 19 (48.72%) patients presented phenotypes of PAIS (PRADER grades 1–5). Thirty patients (76.92%) exhibited cryptorchidism, among which 24 patients exhibited inguinal cryptorchidism, and 6 patients exhibited intra-abdominal cryptorchidism. Only one patient presented intra-abdominal cryptorchidism in the PAIS group. The CAIS group displayed an apparent correlation with cryptorchidism. All PAIS patients exhibited a microphallus phenotype, and 9 of them exhibited hypospadias. The most prominent clinical manifestation of 39 AIS patients was cryptorchidism, and the number of cryptorchidism cases among the CAIS patients was greater than that among PAIS patients (*P* < 0.01) (Table 1). However, there was no significant difference in the incidence of intra-abdominal cryptorchidism between the two groups (Table 2).

**Table 1:** Incidence of cryptorchidism in patients with AIS (*P* = 0.001)

AIS	Non-cryptorchidism	Cryptorchidism	Total cryptorchidism	Ratio
CAIS	0	19	19	100%
PAIS	9	11	20	55%
Total	9	30	39

**Table 2:** Incidence of inguinal cryptorchidism in patients with AIS (*P* = 0.372)

AIS	Inguinal cryptorchidism	Intra-abdominal cryptorchidism	Total cryptorchidism	Ratio
CAIS	14	5	19	73.68%
PAIS	10	1	11	90.9%
Total	23	6	30	

### Hormones

**Fig. 1:**
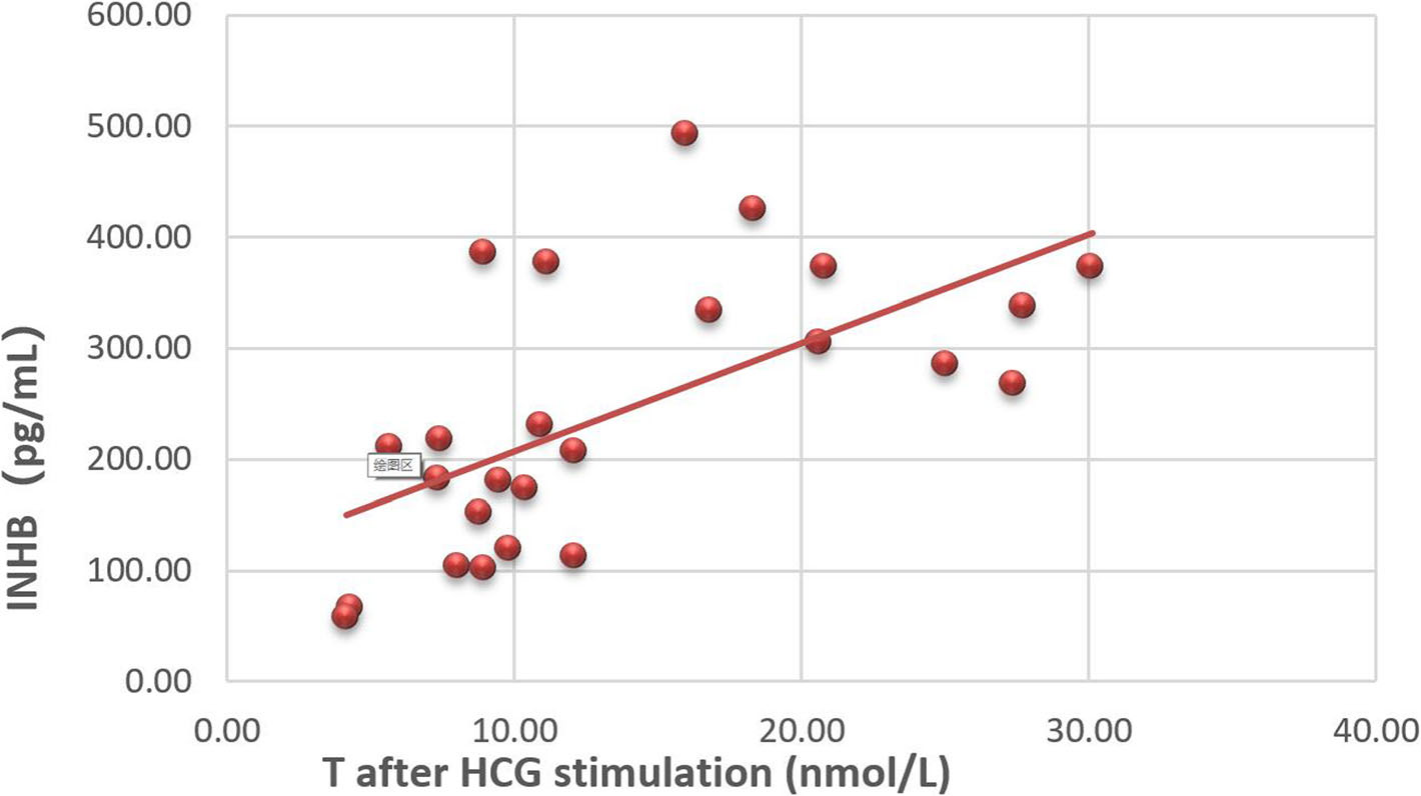
Correlation analysis of INHB and T after HCG stimulation (*r* = 0.618, *P* < 0.01)

**Fig. 2:**
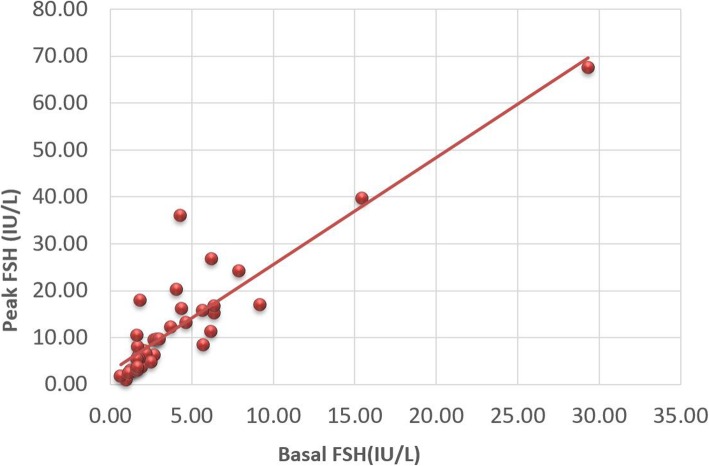
Correlation analysis of basal FSH and peak FSH (*r* = 0.902, *P* < 0.01)

We compared the hormone levels of prepubescent patients under the age of 11 in Tanner stage I, in addition to case C14, in Tanner stage III. Twenty-five of the 39 AIS patients underwent AMH and INHB measurement, which was not performed in the other 14 patients at their own request. The correlation analysis of serum sex hormone levels in AIS patients showed that AMH was positively correlated with INHB (*r* = 0.463) and T after HCG stimulation (*r* = 0.505, *P* < 0.01). INHB was positively correlated with T after HCG stimulation (*r* = 0.618, *P* < 0.01) (Fig. 1). Basal LH was positively correlated with peak LH (*r* = 0.495, *P* < 0.01), basal testosterone (*r* = 0.530, *P* < 0.01) and DHT after HCG stimulation (*r* = 0.488, *P* < 0.01). There was a close positive correlation between basal FSH and peak FSH (*r* = 0.902, *P* < 0.01) (Fig. 2).

**Fig. 3:**
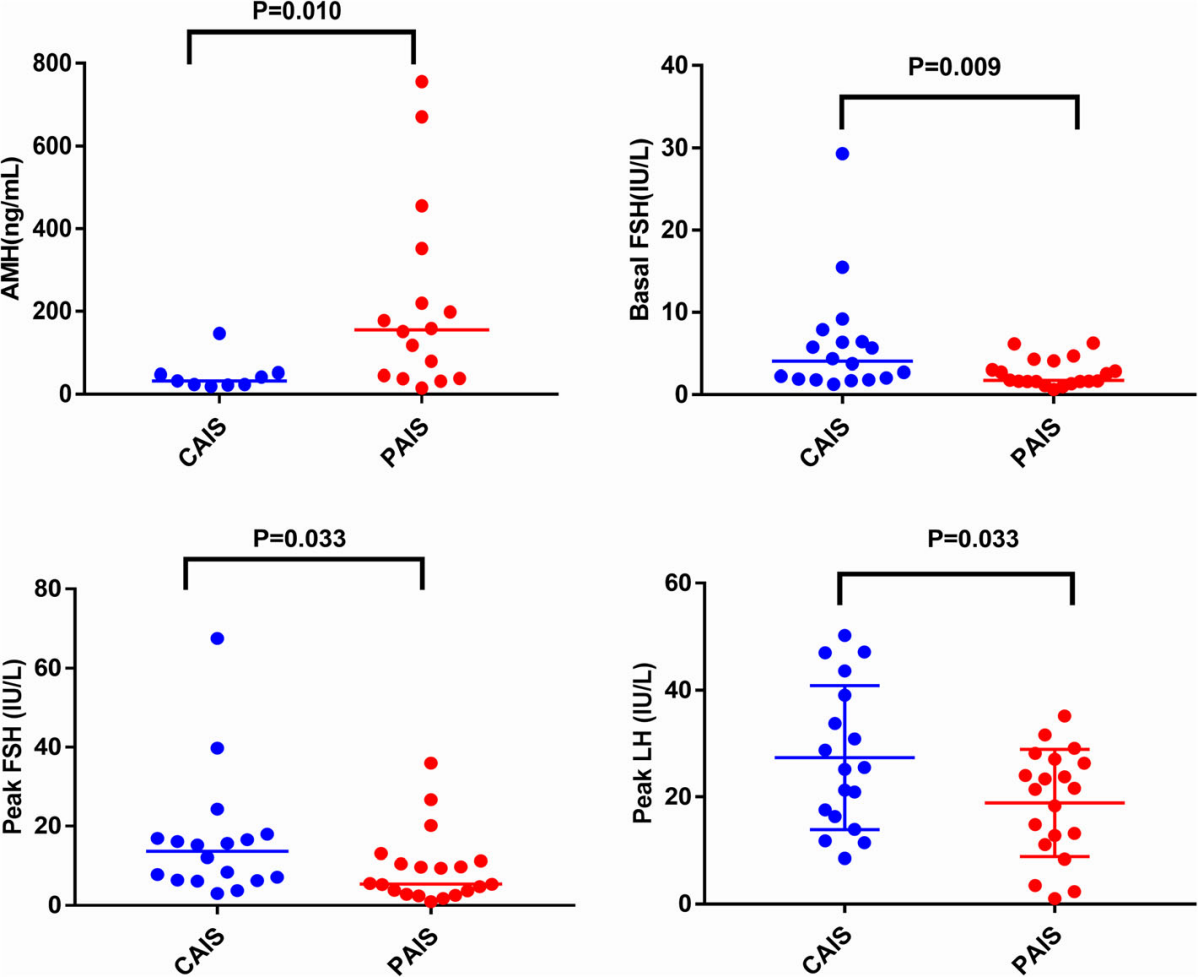
Hormones showing significant differences in the two types of AIS patients

There was no significant difference between the CAIS and PAIS groups regarding the hormone levels of INHB, SHBG, basal LH or basal DHT, the T:DHT ratio, DHT after HCG stimulation or T after HCG stimulation. The hormone levels of AMH (*P* = 0.010), peak LH (*P* = 0.033), basal FSH (*P* = 0.009) and peak FSH (*P* = 0.033) showed statistically significant differences between the CAIS and PAIS groups. The data suggested that CAIS patients presented higher levels of basal FSH, peak FSH, peak LH and lower AMH than PAIS patients (Table 3 and Fig. 3).

**Table 3:** Hormone levels and statistical differences in patients with AIS

Hormones	Group	Median or $\overline{\mathbf{X}}$ ±SD (nmol/L)	Range	N	***P*** value
AMH (ng/mL)	CAIS	32.60	(19.02–146.38)	9	0.010
	PAIS	154.71	(14.77–755.80)	16	
Oestradiol (pmol/L)	CAIS	73	(73.00–110.00)	18	0.303
	PAIS	73	(73.00–202.00)	20	
Basal LH (IU/L)	CAIS	1.18	(0.36–4.93)	18	0.762
	PAIS	1.085	(0.20–5.11)	20	
Basal T (nmol/L)	CAIS	1.105	(0.35–4.80)	18	0.303
	PAIS	0.46	(0.35–8.96)	20	
T after HCG stimulation (nmol/L)	CAIS	10.61	(5.65–20.82)	18	0.072
	PAIS	16.4	(4.15–54.00)	20	
Basal DHT (pg/ml)	CAIS	70.69	(12.16–514.34)	18	0.331
	PAIS	45.4	(12.16–980.23)	20	
DHT after HCG stimulation (pg/ml)	CAIS	255.8	(42.00–491.42)	18	0.762
	PAIS	234.13	(80.57–1006.37)	20	
Basal FSH (IU/L)	CAIS	4.05	(1.29–29.32)	18	0.009
	PAIS	1.735	(0.65–6.26)	20	
Peak FSH (IU/L)	CAIS	13.65	(3.03–67.54)	18	0.033
	PAIS	5.45	(0.99–35.99)	20	
T:DHT ratio	CAIS	8.04	(3.67–34.42)	18	0.228
	PAIS	13.815	(2.74–26.75)	20	
INHB (pg/mL)	CAIS	263.06 ± 33.62	(172.94–424.66)	9	0.548
	PAIS	231.88 ± 33.26	(57.48–493.50)	16	
Peak LH (IU/L)	CAIS	27.39 ± 3.18	(8.51–50.29)	18	0.033
	PAIS	18.86 ± 2.25	(1.02–35.19)	20	
SHBG (nmol/l)	CAIS	122.46 ± 10.82	(22.70–195.70)	18	0.348
	PAIS	108.89 ± 9.39	(35.20–177.70)	20	0.010

(the Mann-Whitney test results are expressed as median values, and the t test results are expressed as the mean and standard deviations, significant differences were considered at *P* < 0.05)

### Molecular studies

Data analysis showed that 21 patients inherited heterozygous AR mutations from their mothers, and the other 5 patients exhibited spontaneous mutations. Most AR gene mutations identified in this study occurred in a ligand-binding domain (LBD) within an exon, and only one patient presented an intron mutation. As shown in Table 4, we found that 29 patients exhibited 21 types of reported pathogenic mutations, and the other 10 patients presented 9 novel AR gene mutations.

**Table 4:** AR gene mutations in 39 AIS patients

AIS	Sex	Age (yr)	Testis position	Mutation	Novel	Exon	Amino Acid	PolyPhen-2	Family history
C1	F	1.3	inguinal region	c.2107 T > C	Y	4, LBD	p. Ser 703 Pro	1.000	Y
C2	F	1.6	abdomen	c.2107 T > C	Y	4, LBD	p. Ser703 Pro	1.000	Y
C3	F	0.4	inguinal region	c.2740C > G	Y	8, LBD	p. Pro 914 Ala	0.609	n.a
C4	F	1.1	inguinal region	c.2351A > G	Y	6, LBD	p. Gln 784 Arg	0.998	n.a
C5	F	2.6	inguinal region	c.2024 T > A,	Y	4, LBD	p.Leu675Gln	0.999	n.a
C6	F	4.4	inguinal region	c.2522G > A	N	7, LBD	p.Arg841His	1.000	Y
C7	F	2.1	abdomen	c.1768 + 1G > C	Y	Intron	Change the shearing mode	U	N
C8	F	2.9	inguinal region	c.2567G > A	N	7, LBD	p. Arg 856 His	1.000	n.a
C9	F	2.3	abdomen	c.1684A > T	Y	2, DBD	p.Ile562Phe	0.999	n.a
C10	F	1	inguinal region	c.2522G > A	N	7, LBD	p.Arg841His	1.000	Y
C11	F	3.8	inguinal region	c.2086G > A	N	4, LBD	p.Asp696Asn	0.999	N
C12	F	3.8	inguinal region	c.2324G > A	N	6, LBD	p.Arg775His	1.000	N
C13	F	1.8	inguinal region	c.2645 T > C	N	8, LBD	p.Leu882Pro	1.000	Y
C14	F	13.7	abdomen	c.2117A > G	N	4, LBD	p.Asn706Ser	0.999	Y
C15	F	2.1	inguinal region	c.2522G > A	N	7, LBD	p.Arg841His	1.000	n.a
C16	F	1.2	inguinal region	c.2290 T>C	N	5, LBD	p.Tyr764His	1.000	Y
C17	F	2.8	abdomen	c.2678C > T	N	8, LBD	p.Pro893Leu	1.000	Y
C18	F	10.8	inguinal region	c.1858 T > C	N	3, DBD	p. Cys620Arg	1.000	Y
C19	F	1.4	inguinal region	c.1415_1416insCGGC	Y	1, NTD	p. Gly 472 fs	U	Y
P1	M	6.8	inguinal region	c.2612C > G	N	8, LBD	p.Ala871Gly	0.999	n.a
P2	M	1	scrotum	c.2521C > T	N	7, LBD	p.Arg841Cys,	1.000	n.a
P3	M	1.5	inguinal region	c.1063G > T	N	1, NTD	p.Glu355Term	U	Y
P4	M	1.9	scrotum	c.2104C > A	N	4, LBD	p.Leu702Ile	0.995	Y
P5	M	1.9	scrotum	C.528C>A	N	1, NTD	p.Ser176Arg	0.842	n.a
P6	M	1.1	scrotum	c.2612C > T	N	8, LBD	p.Ala871Val	0.994	Y
P7	M	1.6	scrotum	c.2531C > A	N	7, LBD	p.Ala844Glu	0.989	n.a
P8	M	0.9	scrotum	c.2531C > A	N	7, LBD	p.Ala844Glu	0.989	n.a
P9	M	1	scrotum	c.2338C > T	N	6, LBD	p.Arg780Trp	1.000	Y
P10	M	9.7	scrotum	c.528C > A	N	1, NTD	p.Ser176Arg	0.842	n.a
P11	M	1,4	inguinal region	c.2221 T > A	Y	5, LBD	p.Ser741Thr	0.555	Y
P12	M	2.4	abdomen	c.1705G > T	N	2, DBD	p.Gly569Trp	1.000	n.a
P13	M	1	inguinal region	c.2567G > A	N	7, LBD	p. Arg 856 His	1.000	n.a
P14	M	1.8	inguinal region	c.2441 T > A	Y	6, LBD	p. Phe814Tyr	0.999	n.a
P15	M	1.1	scrotum	c.2638G > T,	N	8, LBD	p. Asp880 Tyr	1.000	Y
P16	F	1.2	inguinal region	c.2450-42G > A	Y	7, LBD	U	U	Y
P17	F	1.7	scrotum	c.2450-42G > A	Y	7, LBD	U	U	Y
P18	M	6.7	inguinal region	1823G > A	N	3, DBD	p.Arg608Gln	1.000	Y
P19	M	10.7	inguinal region	c.170_172dup	N	1, NTD	p.Leu57dup	U	Y
P20	F	1.3	inguinal region	c.2740C > T	N	8, LBD	p.Pro914Ser	0.996	Y

*F* Female, *M* Male, *n.a.* data not available, *P* Positive, *N* Negative or no, *Y* Yes, *U* Unknown, *LBD* Ligand-binding domain, *NTD* N-terminal domain, *DBD* DNA-binding domain

## Discussion

### Clinical characteristics

T and DHT are important androgens in the human body that act by binding to androgen receptors throughout an individual’s lifespan [7]. Androgen insensitivity results in a series of abnormal characteristics of sexual development. Although the effect of AMH is normal in AIS patients and they do not have female internal genitalia, such as a uterus or ovaries, T and DHT cannot effectively promote the development of the Wolffian ducts or genitourinary sinus [8]. These patients may exhibit sexual dysplasia, blind vagina, cryptorchidism, microphallus or hypospadias [9]. Cryptorchidism was the major phenotype observed in this study, which was characterized as inguinal cryptorchidism in most cases. Intra-abdominal cryptorchidism showed a higher incidence in CAIS patients than in PAIS patients. Inguinal cryptorchidism was more common because androgens can drive testicular descent into the inguinal region. The external genitalia of the PAIS patients were ambiguous, and these patients exhibited microphallus with or without hypospadias [10]. However, hypospadias is not sufficient for the identification of AIS and other 46, XY DSDs. In 46, XY DSD patients with cryptorchidism and a female phenotype, after excluding complete gonadal dysplasia, 17α-hydroxylase and 17β-HSD type 3 deficiency and interstitial cell dysplasia, it is necessary to perform the diagnosis of AIS.

### Hormones

According to basal hormone levels, prepubescent AIS patients might exhibit higher T or E2 levels. There was no significant difference in T or E2 levels between the PAIS group and the CAIS group. The basal T level may present a characteristic increment in AIS patients since most patients with 46, XY DSD are diagnosed with male dysfunction with a decrease in T. According to previous studies, AIS patients exhibit higher E2 levels in adulthood [11]. This study suggested that AIS patients might present a nondetectable elevation of serum oestradiol in prepuberty. In addition, AIS patients may show breast development at puberty.

Consistent with previous studies, we found that the LH level increased dramatically after GnRH stimulation in AIS patients, indicating that a large amount of LH was stored in the anterior pituitary [12]. Our data demonstrated that CAIS patients presented higher levels of FSH and peak LH than PAIS patients. The peak LH levels of CAIS (27.39 ± 3.18 IU/L) and PAIS (18.86 ± 2.25 IU/L) patients showed an evident increase compared with those of healthy children, and their basal LH levels were approximately 23 times or 17 times higher than those in healthy children. However, peak FSH levels exhibited a weak response to GnRH stimulation, reaching levels approximately 3 times higher than the basal FSH level. These data suggested that peak LH levels in AIS patients show a higher correlation with androgen resistance and influence the severity of AIS in prepubescent children.

It is worth noting that the T level after HCG stimulation was lower in CAIS patients than in PAIS patients, while the DHT level after HCG stimulation was higher than that in PAIS patients. We suspected that T might be converted into more bioactive DHT by the 5α reductase 2 enzyme in the CAIS group, which showed stronger androgen resistance to counter-receptor defects. In male infants with CAIS within the few months after birth, T levels do not increase after HCG stimulation, whereas the T levels of male infants with PAIS present a spontaneous increase, which could indirectly support the findings our study [13].

AMH and INHB are biomarkers of the testis that are secreted by Sertoli cells which are activated in infancy and childhood [14]. AMH and INHB play important roles in gonadal development and sex differentiation and have discriminating effects on patients with impalpable testis [15–17]. The data showed no significant difference in INHB levels between the two groups distinguished in this study, while there was a significant difference in AMH levels (*P* = 0.010). CAIS patients exhibited lower concentrations of AMH than PAIS patients, suggesting that AMH is crucial for the classification of AIS. Compared with the reference range reported in the literature [18], the median hormone level of AMH within the CAIS patients was within the normal range, while the median hormone level of AMH within the PAIS patients was above the normal range. These results may be due to the combined regulation of FSH and androgen.

The PAIS patients presented higher T:DHT ratios and SHBG concentrations than the CAIS group, but these differences were not significant. The T:DHT ratios of case C8 and case C16 were above 30, although we did not find any mutations in these patients via SRD5A2 gene sequencing. These results suggested that the T:DHT ratio cannot be used alone to differentiate AIS from other 46, XY DSDs. The T:DHT ratio in most AIS patients was lower than in typical cases of SRD5A2 deficiency [19]. In addition, this ratio may be higher than 30 a small number of AIS patients. The serological manifestations of prepubescent AIS patients were somewhat similar to those in early adolescence or puberty. An increase in LH secretion contributes to T secretion, and T can be converted into DHT by the SRD5A2 enzyme or into oestradiol by the aromatase enzyme.

### Genetic analysis

Mutations in the AR gene can lead to AIS, and AIS requires genetic diagnosis with or without a family history of AIS because the rate of spontaneous mutation is 30% [20]. Common mutations of the AR gene include missense mutations, insertions and deletions. Most of these mutations are relevant to AIS, and few are related to gonadal tumours. Consistent with international research findings, the most common mutations identified in this study were missense mutations, and exon 7 was the exon that was most prone to mutation. The majority of patients exhibited AR gene missense mutations, including 1 mutation that led to the termination of protein synthesis. Other kinds of mutations caused different phenotypes, including transcriptional shear, frame shift mutations and gene duplications.

Cases C1 and C2, carrying the c.2107 T > C (p. Ser703Pro) mutation, came from the same family. Their mutations were inherited from their mother and exhibited pathogenic potential according to PolyPhen-2 analysis. This mutation was not reported previously. However, there were some similar reports in the McGill mutation database; for example, the c.2107 T > G (p. Ser703Ala) mutation of the AR gene causes a complete female phenotype [21]. c.2740C > G (p. Pro 914 Ala) was indicated to cause CAIS in this study, and this mutation has not been reported previously. Studies have indicated that similar amino acid changes, including the c.2740C > T (p. Pro914Ser) mutation, can cause PAIS, which was also found in case P20 in this study [20]. The previously reported c.2450-42G > A mutation creates an alternative splice acceptor site in intron 6 of the AR gene, which could lead to the production of a large number of abnormal mRNAs and a relatively small number of wild-type mRNAs, resulting in AR protein dysfunction [22].

Four patients, including 3 CAIS patients and 1 PAIS patient, presented the c.2522G > A (p. Arg841His) hot spot mutation in this study. This mutation results in a broad range of phenotypes from a completely female phenotype to male phenotypes with minor degrees of undervirilisation [23], and the majority of these cases are diagnosed as CAIS. There were no specific correlations between genotypes and phenotypes identified in the AIS patients. Identical AR mutations can lead to variable phenotypic expression because one mutation can produce different phenotypes and appear in different individuals within a family. Forty-five allelic variants that may result in different phenotypes are currently recorded in the McGill AR mutation database. There are no available qualitative data on penetrance at present. The variable phenotypic expression of particular mutations may be due to differences in affected individuals such as somatic cell embedding [24–26].

In this research, we identified 21 reported pathogenic AR gene mutations in 29 patients, and we detected 9 novel mutations, including c.2107 T > C (p. Ser703Pro), c.2740C > G, c.2351A > G (p. Gln784Arg), c.2024 T > A (p. Leu675Gln), c.1684A > T (p. Ile562Phe), c.2221 T > A (p. Ser741Thr) and c.2441 T > A (p.Phe814Tyr). According to the GU-AG rule, the c.1768 + 1G > C mutation was estimated to be a pathogenic mutation on the basis of a change in the corresponding intron to shearing mode, which is genetically consistent with the pathogenic pattern of X-linked recessive inheritance. c.1415_1416insCGGC (p. Gly472fs) is a novel frameshift mutation identified the AR gene. Studies have shown that a 2-base deletion and frameshift mutation at this site can lead to CAIS [27]. Mutations including c.2024 T > A (p. Leu675Gln), c.2351A > G (p. Gln784Arg), c.1684A > T (p. Ile562Phe), c.1768 + 1G > C and c.1415_1416insCGGC (p. Gly472fs) cause a complete female phenotype, while the c.2441 T > A (p. Phe814Tyr), and c.2221 T > A (p. Ser741Thr) mutations lead to PAIS. The 9 novel AR mutations identified in our study cause different types of AIS. These findings will provide deeper insight into AIS and contribute to its clinical assessment.

## Conclusions

Abnormalities in the AR gene can cause AIS, with phenotypes ranging from CAIS with a complete female phenotype to PAIS with minor degrees of undervirilisation or infertility. In this research, cryptorchidism showed a higher incidence in CAIS than PAIS patients. We demonstrated that AIS patients could not be distinguished solely by their hormone levels of E2, INHB, SHBG, basal LH, basal T, or basal DHT, the T/DHT ratio, T after HCG stimulation or DHT after HCG stimulation. However, CAIS patients exhibited higher hormone levels of basal FSH, peak FSH, and peak LH and lower AMH levels than PAIS patients. We identified 21 reported pathogenic mutations and 9 novel mutations of AR genes that could result in different AIS symptoms. These findings provide deeper insight into AIS diagnosis and will contribute to its clinical assessment.

## Data Availability

All data generated or analysed during this study are included in this published article. The datasets used and/or analysed during the current study are available from the corresponding author upon reasonable request.

## Abbreviations

AR: Androgen receptor
AIS: Androgen insensitivity syndrome
CAIS: Complete androgen insensitivity syndrome
PAIS: Partial androgen insensitivity syndrome
DSD: Disorders of sex development
INHB: Inhibin B
SHBG: Sex hormone-binding globulin
LH: Luteinizing hormone
FSH: Follicle-stimulating hormone
T: Testosterone
DHT: Dihydrotestosterone
E2: Oestradiol
HCG: Human chorionic gonadotropin
AMH: Anti-Mullerian hormone
GnRH: Gonadotropin-releasing hormone
LBD: Ligand-binding domain
NTD: N-terminal domain
DBD: DNA-binding domain
